# Immature Stages of Development in the Parasitoid Wasp, *Diachasmimorpha longicaudata*

**DOI:** 10.1673/031.010.5601

**Published:** 2010-06-06

**Authors:** Leonela Zusel Carabajal Paladino, Alba Graciela Papeschi, Jorge Luis Cladera

**Affiliations:** ^1^ Laboratorio de Genètica de Insectos de Importancia Económica IGEAF INTA Castelar, CP 1712, Buenos Aires, Argentina; ^2^ Laboratorio de Citogenética y Evolución, Departamento de Ecología, Genética y Evolución, FCEN, UBA, CP C1428EGA, Buenos Aires, Argentina

**Keywords:** Hymenoptera, developmental stages, superparasitism

## Abstract

The morphological changes experienced during the immature stages of the solitary parasitoid *Diachasmimorpha longicaudata* (Ashmead) (Hymenoptera: Braconidae: Opiinae) were studied. This natural enemy of several species of tephritid fruit flies is widely used in biological control strategies. Immature stages are poorly understood in endoparasitoids because they exist within the host. In the present work, developmental processes are described for this species, reared in *Ceratitis capitata* (Wiedemann) (Diptera: Tephritidae) larvae under controlled environmental conditions. At 25° C, 85% RH, and with an 18:6 L:D photoperiod, preimaginal development takes about 16 days. Five preimaginal stages can be described: egg, three larval instars, prepupa, pupa, and pharate adult. Superparasitism was found in 20% of the host pupae, and the number of oviposition scars was positively correlated with the number of parasitoid larvae per host puparium. The results are compared and discussed with previous studies on related species.

## Introduction

Using hymenopteran parasitoids as biological control agents in any integrated pest management program largely depends on how well the natural history is known for the particular group of species. But, despite the importance of the subfamily Opiinae in biological control strategies, the morphological changes it experiences during the immature stages have been poorly studied because preimaginal development takes place inside the host.

*Diachasmimorpha longicaudata* (Ashmead) (Hymenoptera: Braconidae) is an obligate endoparasitoid of third instar larvae of fruit flies (Diptera: Tephritidae), although some studies indicate that it can occasionally develop inside other Dipterans, such as *Musca domestica* (Terán López 1983). It is native to Malaysia, India, New Britain, Borneo, Saipan, and the Philippine Islands (Bess 1961; Clausen et al. 1965).

*D. longicaudata* is the most important parasitoid species that is used as part of integrated pest management programs against fruit flies of the genera *Bactrocera, Anastrepha* and *Ceratitis,* comprising some of the world's most widespread and damaging pests of fruticulture and horticulture and causing enormous economic losses (Cancino Díaz et al. 1993; Malavasi et al. 1994; Ovruski et al. 1999). This parasitoid has been released and successfully established in Hawaii, Australia, Fiji, Mexico, Costa Rica, and Trinidad (Clausen 1978; Wharton et al. 1978; Wharton 1989). However, its biology is still not understood well enough to analyze its impact on exotic ecosystems and to improve its production in mass rearing facilities.

*D. longicaudata* females usually lay only one egg inside the host larva, but when hosts are scarce, and under laboratory conditions, superparasitism occurs as more than one egg can be deposited in host larvae (Greany et al. 1976). However, only one individual from each puparium reaches the adult stage. A previous study carried out by Montoya et al. (2000) on *D. longicaudata* reared in *Anastrepha ludens* (Loew) (Diptera: Tephritidae) indicated that there was a highly positive correlation between the number of scars per puparium and the number of first instar parasitoids per puparium.

Adult *D. longicaudata* usually emerge from fruit fly puparia a few days after the emergence of adult flies from unparasitized puparia and, depending on temperature and humidity during development, parasitoid males emerge between two and three days before parasitoid females (Hoy 1994; Bautista et al. 1997). A preliminary study showed that male parasitoids emerge around 17 days post parasitism (DPP) when reared at 25° C and 75% relative humidity (Carabajal Paladino et al. 2005).

The aim of this study was to thoroughly describe the developmental time, survival rates and morphology of the immature stages of *D. longicaudata* reared in *Ceratitis capitata* (Wiedemann) (Diptera: Tephritidae) larvae under controlled environmental conditions and to compare the results with previous studies of related Opiinae species (Pemberton et al. 1918; Willard 1920; Biliotti et al. 1959; N'Guetta 1990; Hurtrel et al. 2001; Rocha et al. 2004). This information contributes to the ecological, biological and genetic characterization of the species to optimize the use of *D. longicaudata* as a biological control agent.

## Materials and Methods

### Experimental insects

Adult *D. longicaudata* were imported from Mexico to Tucumán (Argentina) in 1998 and were introduced to our laboratory in 2001 (SENASA, exp n° 14054/98). They were maintained in glass flasks with water and honey. Larvae of *C. capitata* were reared in a larval medium (Terán 1977) and offered to adult *D. longicaudata* females in plastic Petri dishes (5 cm in diameter and 1 cm in depth) covered with voile fabric for a period of 4 to 6 hours. The larvae were then transferred into a plastic tray with fresh artificial larval medium, where they completed their development. The trays were placed over a thin layer of vermiculite, which acted as a pupation medium. Both larvae and pupae were maintained in an incubator at 25° C, 85% RH and an 18:6 L:D photoperiod.

### Experimental procedures

To follow development of *D. longicaudata,* third instar larvae of *C. capitata* were exposed to 7-day-old adult females of *D. longicaudata,* according to the procedure described above for a period of 4 hours. This short period was used for a better synchronization of stage duration. The exposed hosts were then maintained under standard rearing conditions.

The development of *D. longicaudata* was followed during 20 days which was considered long enough to fully cover the development of all individuals.

It is not possible to follow individual eggs throughout their development due to the endoparasitic characteristic of *D. longicaudata.* To study the immature stages of development of the parasitoid, 20 homogenous samples were obtained from the exposed material. During the first and second days, the host is still a larva and tends to cluster in the rearing tray, so it was difficult to obtain enough homogeneous samples to dissect. To overcome this problem, three spoonfuls of the larval medium containing the larvae previously exposed to the parasitoids were randomly sampled during the first two days, and every host larvae was recovered. By the third day all of the *C. capitata* larvae reached the pupa stage. Pupae were recovered from the pupating medium and divided into 18 groups, all of which were kept in an incubator under the study conditions. Every 24 hours, one of the groups was dissected under a stereoscopic binocular microscope, and the parasitoids were photographed. For every parasitoid found, the stage of development and other characteristics (presence of fungus, color, etc.) were recorded. The recorded data were plotted to obtain the developmental curves for each instar. The duration of each instar was estimated as the time between its developmental curve and the next one, at a frequency of 0.5.

Superparasitism was recorded by counting the number of oviposition scars (marks left in the host's cuticle after being pierced by the female parasitoid with its ovipositor) and the number of parasitoids found inside the puparium on the second and third days after parasitoid attack.

### Statistical analysis

The correlation between the number of scars and the number of parasitoid larvae per host puparium was analyzed using the non-parametric Spearman test, since these variables have a Poisson distribution. The independence of the variables was studied by a χ2 test, including the data from the pupae with 1 to 6 scars and 0 to 3 parasitoids, which were the bulk of the cases (95.3%).

A “Difference test” was performed in order to compare the statistical significance of the differences between the correlation coefficient obtained in the present study and the one presented by Montoya et al. (2000), where *D. longicaudata* was reared in *A. ludens.*

## Results

### Developmental analysis

Between 665 and 853 larvae or pupae of *C.* *capitata* were dissected daily, and 119–212 parasitoids were found and examined each day. Cases of superparasitism were not included in the developmental analysis. Overall parasitism was 20%. The number of individuals found daily in each stage, either alive or dead, is summarized in Table 1.

**Figure 1.**
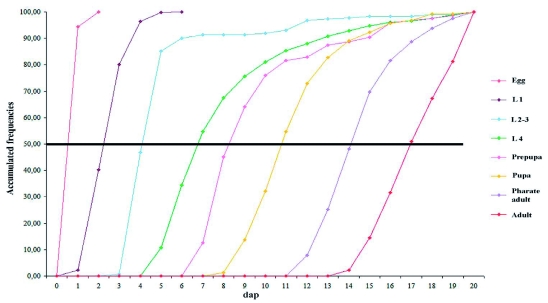
Developmental curves of *Diachasmimorpha longicaudata.* The frequency of live individuals in the different stages is plotted against the days after parasitism. L1: First instar larva, L2: Second instar larva, L3: Third instar larva, dap: days after parasitism. High quality figures are available online.

The complete immature development of this species, from egg to the emergence of the adults, took about 16 days (at 25° C, 85% RH and 18:6 L:D photoperiod). The duration of each stage of development was as follows: egg = 1.5 days, first instar larva = 1.5 days, second instar larva = 2.7 days, third instar larva = 1.5 days, prepupa = 2.5 days, pupa = 3 days, and pharate adult = 3 days (Table 1, Figure 1).

### Morphology

**Figure 2.**
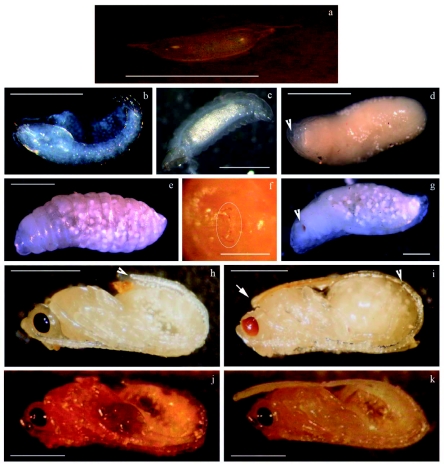
Immature stages of development *of Diachasmimorpha longicaudata.* (a) Egg, Bar: 500 µm, (b) First instar larva; (c) Late first instar larva; (d) Second instar larva, arrowhead points to the mouth; (e) Third instar larva; (f) Mandibles of third instar larva, Bar: 500 µm; (g) Prepupa, arrowhead points to the pigmented eyes; (h) Male pupa, arrowhead points to the end of the antennae; (i) Female pupa, arrowhead points to the end of the antennae, arrow shows the ovipositor; (j) Male pharate adult; (k) Female pharate adult. Bar: 1 mm, unless otherwise noted. High quality figures are available online.

**Egg** (Figure 2a): One day after parasitization eggs were slightly cylindrical, translucent, and very difficult to visualize amidst the host tissue. The few eggs found on the second day were brighter and more swollen.

### Larva:

**Figure 3.**
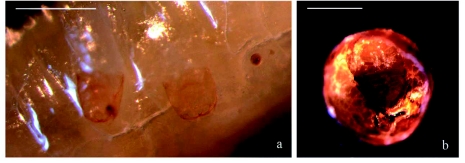
(a) Chitinized heads of two first instar *Diachasmimorpha longicaudata* larvae visualized through *Ceratatis capitata* larva's cuticle, (b) Dead second instar *D.* *longicaudata* larva at the bottom of a section of a *C.* *capitata's* puparium. Bar: 1 mm. High quality figures are available online.

***First instar larva*** (Figures 2b, 2c): The head was large, highly chitinized, and bore a pair of sickle-like mandibles that could usually be seen through the host larval cuticle (Figure 3a). The body was translucent and segmented, but it becomes whitish as the digestive canal became gradually filled and swollen with globules of fat.

***Second instar larva*** (Figure 2d): When the parasitoid larva reached the second larval instar, the molted skin was easily dissected from the fly puparium. The head could not be differentiated from the other body segments, and the mandibles were translucent and difficult to visualize (Figure 2d, arrowhead). The body was entirely glabrous, and the segments were less distinguishable than in the previous instar.

Approximately 10% of the dissected individuals remained in second-third larval instar from the sixth day after parasitization, but they were mostly dead and dehydrated at the bottom of the puparium (Figure 3b).

***Third instar larva*** (Figure 2e): The parasitoid was much bigger and filled almost the entire host puparium. Pointed mandibles with brownish chitinizations at the tips and bases were distinguished on the head (Figure 2f). The body was yellowish, well-segmented, and large white cells were clearly visible under the integument. The larvae could move from side to side and change its orientation inside the puparium. A dark oval meconium could be seen through the cuticle at the caudal end. This meconium contained the digestive waste and was eliminated during the adult emergence (Cancino Díaz 1998).

***Prepupa*** (Figure 2g): The prepupa was characterized by a lack of mobility, compared to the third larval instar, and the beginning of a reddish pigmentation of the eyes (Figure 2g, arrowhead). The development of the pupa could be seen through the cuticle as this instar proceeded. No visible changes in the mandibles' morphology were detected.

**Table 1.**
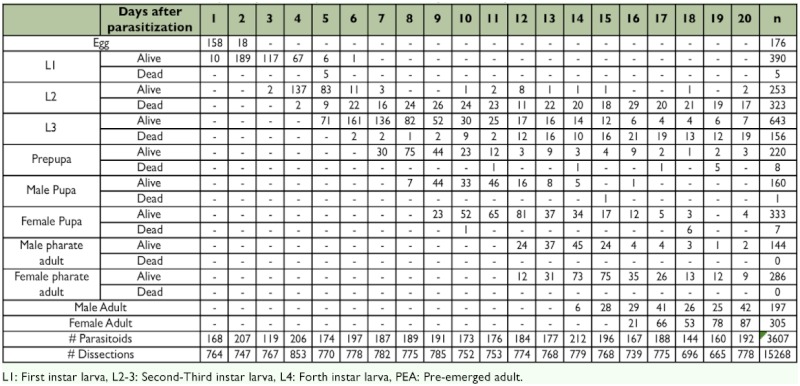
Number of individuals of each preimaginal developmental instar of *D.* *longicaudata* daily dissected.

**Pupa** (Figures 2h, 2i): The shape of the pupa was similar to that of an adult, with a white body and fully pigmented red eyes. As pupation progressed, both the eyes and the body became darker. Sexual dimorphism was very noticeable. Females presented a well-developed ovipositor bent to the dorsal side (Figure 2i, arrow), and males had valves that corresponded to the external part of the reproductive system at the ventral-caudal region. Furthermore, the antennae were noticeably longer in males than in females (Figures 2h and 2i, arrowheads).

***Pharate adult*** (Figures 2j, 2k): The only difference from the pupa was that the body started to acquire a brownish pigmentation, as the sclerotization process took place.

### Superparasitism analysis

A total of 297 pupae were analyzed, and the results are summarized in Table 2.

When the number of scars was impossible to count, the situation was recorded as “present,” and when it was difficult to determine the presence of oviposition, it was recorded as “uncertain.”

The number of oviposition scars ranged from 0 to 12, with the mode at one scar (94 cases, 31%). Host pupae with more than six scars were very infrequent (9 cases, 3%). The number of parasitoid larvae inside each puparium ranged from 0 to 9. Superparasitism was found in 19.87% of the analyzed host pupae.

**Table 2.**
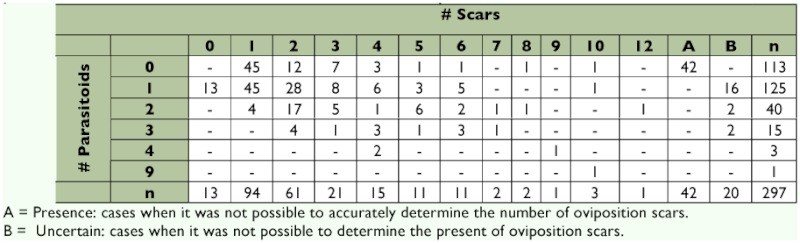
Number of scars found per puparium, number of parasitoids dissected by puparium and number of cases that presented the different combinations of the number of scars and parasitoids.

The correlation analysis between the number of scars and the number of parasitoid larvae per host puparium showed a significant positive correlation (Spearman R = 0.402, p < 0.001, *n* = 297). The results of the χ^2^ test confirmed the non-random distribution of these variables (p < 0.001).

The Difference test performed between the current data and the data obtained by Montoya et al. (2000) (r = 0.9492, p < 0.001, n = 100) showed a significant difference between the two studies, which led to the conclusion that the relation between number of scars and the number of parasitoid larvae per host puparium is stronger when *D. longicaudata* is reared in *C. capitata* than when it is reared in *A. ludens.*

## Discussion

In the present study the morphological changes occurring to *D. longicaudata* during its development inside the host's puparium were analyzed, along with the duration of each stage under controlled environmental conditions. The preimaginal development of this species took about 16 days (at 25° C, 85% RH and an 18:6 L:D photoperiod) when reared in *C.* *capitata.*

The size and duration of parasitoid developmental stages varied with the size, age and quality of the host in which they were reared (Lawrence et al. 1976; Lawrence 1990) as well as the environmental conditions at which they were kept. Hurtrel et al. (2001) analyzed the developmental time of *Diachasmimorpha tryoni* (Cameron) (Hymenoptera: Braconidae) reared in *C.* *capitata* larvae at 25° C, 85% ± 15 % RH and a 12:12 L:D photoperiod, and found that male and female development took 19.80 days and 21.47 days, respectively. Those times were longer than when observed for *D. longicaudata* possibly because of interspecific differences, although some effect of the relative humidity or the photoperiod cannot be ruled out.

These observations of the morphology and characteristics of the immature developmental stages in *D. longicaudata* are in accordance with previous reports on related species of Opiinae parasitoids, such as *D. tryoni, D. fullawayi, Opius humilis, Psyttalia concolor, P. fletcheri,* and *Fopius arisanus* (Pemberton et al. 1918; Willard 1920; Biliotti et al. 1959; N'Guetta 1990; Hurtrel et al. 2001; Rocha et al. 2004).

According to Chapman (1998), an insect is referred to as a prepupa during the period of quiescence that occurs before the ecdysis to a pupa. Other authors also differentiate the stage pharate adult (Aluja et al. 1998; Kitthawee et al. 1999). Although they may not be considered true developmental stages, as no real molt occurs before them, they have specific morphological traits that allow their identification, and they have proven to be very useful for other types of studies, such as cytogenetic analysis (Kitthawee et al. 1999). Therefore, seven preimaginal stages were considered in *D. longicaudata:* egg, three larval instars, prepupa, pupa, and pharate adult.

First instar *D. longicaudata* larvae were found in *C. capitata* larvae, suggesting that the egg may hatch while the host is still in active feeding larval stage. This observation was also made by Pemberton and Willard (1918) for *D. triony, D. fallowayi* and *O. humilis.* The parasitized host larva continues to feed and develops to maturity forming a perfect puparium, but then the complete histolysis of the larval tissues occurs within the puparium. Hence, its content is a liquid mass containing the rapidly developing parasite larva (Pemberton et al. 1918). During the development of an endoparasitic insect, various metabolic and endocrinological changes occur. Some parasitoid species regulate host development to satisfy their own physiological requirements (Vinson et al. 1980; Lawrence 1982). However, it is also known that, sometimes, the development of the endoparasitoid is tied to the endocrine events associated with the host metamorphosis, while others may exhibit a more facultative synchronization between their development and that of their host (Lawrence 1986). When *D. longicaudata* is reared in *Anastrepha suspensa* larvae, it never molts until the onset of larval-pupal apolysis of its host, indicating that the parasite utilizes endocrine signals from the host to mark its change in nutritional quality (Lawrence 1982, 1986). The temperature and humidity of the rearing conditions also affect the parasitoid development, indirectly, by accelerating or decreasing the developmental rate of its host; and directly, by affecting its own metabolic rate during the last stages, when the host is already eaten.

Under the stereoscopic binocular microscope, the second and third instar larvae, previously described by other authors, were impossible to distinguish. According to Pemberton and Willard (1918), the only differences between the second and the third larval stages are the molt of mandibles and an increase in size. The mandible molt was not observed, and the difference in size was not reliable enough since the parasitoid larval size depends directly on a number of factors, including the size and nutritional state of the larval host when it was parasitized, environmental conditions, whether the parasitoid is diapausing (diapausing individuals are usually smaller than nondiapausing ones), etc. Furthermore, according to the results, the short period between the first and third larval instars does not seem enough for two prolonged larval instars to occur. For practical purposes, it would be better to consider the second and third larval instars as one category (second instar larvae in the present work), as it has been suggested for other parasitoid species such as *D. tryoni* and *F. arisanus* (Hurtrel et al. 2001; Rocha et al. 2004).

The live individuals in early stages of development regularly found from the fourth day after parasitization onward can be explained by the presence of diapausing parasitoids. It has been shown that *D. longicaudata* has a diapause period during the fourth larval instar or during prepupa, which depends on the temperature, humidity, photoperiod, host and host plant (Clausen et al. 1965; Ashley et al. 1976; Aluja et al. 1998; Carvalho 2005).

This study shows that *D. longicaudata* males and females have different developmental rates, as can be observed by the differences in the pigmentation found between individuals of both sexes of the same age. Male pupae could be identified one day before female pupae, and adult males emerged from their puparium two days before females. As it is difficult to differentiate both sexes before the pupa stage (at least with the methodology used in this study), it is not possible to confirm whether the eggs hatch at different times or if males accelerate or females delay their development at a more advanced stage. The observation that the sex ratio is biased toward females is currently under investigation.

As no normally developed fly was found in parasitized puparia, it was interesting to find perfectly formed *C. capitata* pupa inside puparia with oviposition scars (113 cases, 38.05%), which are usually used as a sign of parasitism. Although a significant correlation was found between the number of oviposition scars and the number of parasitoids found inside the puparium, the former does not indicate directly the number of parasitoids that will be found. These facts will have to be taken into account in further studies of superparasitism and parasitism rates, among others.

It was common to observe superparasitized host pupae. As it was stated in the introduction, only one individual is able to complete development. Many theories have been suggested to explain the process that affects the survival of the surplus larvae. They include cannibalism, physical attacks between larvae followed by encapsulation of the injured one, and physiological suppression, where the level of oxygen inside the host or chemical compounds segregated by the older larva do not allow the development of the surplus larvae (Fisher 1963; Lawrence 1988b; Vinson et al. 1998). Lawrence (1988a) studied the interactions between first instar larvae of *D. longicaudata in vivo* and *in vitro,* reporting that both physical attack and physiological suppression occur in this species. The present observations agree only with the latter, as no signs of physical injury were detected, and while one larva was always found to be in advanced first or second-third instars, the others remained intact in early first instar and eventually died. The same observation was made by Montoya et al. (2000) when studying superparasitism in *D. longicaudata* reared in *A. ludens.*

The description of the changes that take place inside the puparium during the development of *D. longicaudata,* as well as other characteristics found during the dissections, contribute to a better knowledge of the biology of the species. All this information will be useful to determine when release has to be done to minimize the time the parasitoid has to be kept under rearing conditions, and to estimate the intrinsic rates of population increase, which is important, for example, to establish the releasing methods in the control programs, either inoculative, seasonal inoculative or inundative release (Lewontin 1965; van Lenteren 1986). This information will also provide the necessary knowledge for cytogenetic studies, for the design of effective quality control tests for this natural enemy, and for studies related to the differential impact of radiation or other mutagenic sources during early stages of development.
